# Tobacco consumption and nicotine dependence in Bengo Province, Angola: A community-based survey

**DOI:** 10.1371/journal.pone.0188586

**Published:** 2017-11-27

**Authors:** João M. Pedro, Miguel Brito, Henrique Barros

**Affiliations:** 1 CISA—Centro de Investigação em Saúde de Angola, Caxito, Angola; 2 EPIUnit, Instituto de Saúde Pública, Universidade do Porto, Rua das Taipas, nº 135, Porto, Portugal; 3 Escola Superior de Tecnologia da Saúde de Lisboa, Instituto Politécnico de Lisboa, Av. D. João II, Lote 4.69.01, Lisboa, Portugal; 4 Faculdade de Medicina, Universidade do Porto, Al. Prof. Hernâni Monteiro, Porto, Portugal; Ruhr-Universitat Bochum, GERMANY

## Abstract

There is concern about the potentially increasing use of tobacco in Angola. However, information on the frequency and determinants of this use is not systematised. This study aimed to estimate the prevalence of tobacco consumption and nicotine dependence among smokers in an Angolan population and considering individual socio-demographic and behavioural characteristics. A community-based survey with 2,472 respondents (age range: 15–64 years) was conducted in 2013–2014 in the country’s Bengo Province. The collection methodology for assessing each type of tobacco consumption and its daily quantification followed the World Health Organization STEPwise approach to chronic disease risk factor surveillance. The Fagerström Test for Nicotine Dependence was also used to assess smokers. Mean values for prevalence of tobacco use and nicotine dependence were estimated by sex and by previously defined variables. Daily smoking (6.1%) was found to be higher for males (10.0%) them among females (2.6%), and the amount of ex-smokers (7.5%) was higher them smokers. Only 0.2% of those surveyed reported use of smokeless (chewing) tobacco. One-third of ever-smokers reported having started smoking daily before age 18. Nicotine dependence levels were classified as very low or low in 83.6% of the smokers. Daily smoking prevalence increased with age, and was higher in rural areas and among individuals with no formal education, lower incomes, and alcohol consumption. This population presented a low smoking prevalence, along with a low number of daily smoked cigarettes and low levels of nicotine dependency, despite the low prices of, and easy access to, manufactured cigarettes. These two factors conjugated with the current absence of an Angolan policy for tobacco control, enhance the susceptibility for rising overall tobacco use in the near future.

## Introduction

Tobacco is a major risk factor for multiple non-communicable diseases (NCDs), including cancer, chronic lung diseases, and cardiovascular diseases. Tobacco use presently accounts for around seven million deaths every year worldwide [1], and this is projected to increase to eight million by 2030 [2]. A 30% relative reduction until 2025 in the prevalence of current tobacco use in people aged 15 years or older is one of the nine voluntary global targets defined to reduce the preventable and avoidable burdens of morbidity, mortality, and disability due to NCDs [3].

Low- and middle-income countries are facing increased in tobacco use, while the opposite is occurring in high-income countries [4,5,6]. It is also estimated that nearly 80% of the world’s tobacco smokers are from low- or middle-income countries [7].

Although information on the prevalence, trends, patterns, and determinants of tobacco use is still limited for sub-Saharan Africa [8,9], there is evidence of increasing use in some countries [9]. Smoking is expected to increase in the region as a result of the tobacco industry’s strategy of creating new consumers to offset the declines in traditional markets [10,11].

In Angola, which ratified the Framework Convention on Tobacco Control in 2007, there is no national survey on tobacco consumption, and the defined policies in the convention that aim at effective tobacco prevention and control have not been fully implemented, especially regarding smoke-free environments, taxation, health warnings on tobacco product packages, and bans on advertising, promotion, and sponsorship of such products [12,13,14]. The leading causes of mortality and morbidity in Angola are communicable, maternal, neonatal, and nutritional diseases, but the rates of premature deaths due to NCDs are rising [15]. The urbanization process and westernization of life habits in Africa is followed by increased exposure to behavioural risk factors for NCDs, such as alcohol consumption and tobacco use [3,4].

Tobacco, one of Angola’s major industries, was established in the country during the Portuguese Colonial War (1961–1974) and has experienced 400% growth since 2002, although there are no tobacco plantations or manufacturing industries in the country [16]. The going market price for a pack of 20 cigarettes is around $1, and since 2009, British American Tobacco has held a monopoly in the country’s tobacco industry [14,16].

There is concern about the increasing use of tobacco in Angola following rapid economic growth, given the low prices for tobacco products and in the absence of restrictive legislation or organized health promotion campaigns. However, there is scarce information on the frequency and determinants of tobacco use in representative samples, further limiting health plan efforts and evaluation of interventions. The present study is the first report on the use of tobacco and the level of nicotine dependence among smokers in a large Angolan community sample of people aged 15–64 years.

## Materials and methods

Results presented herein were extracted from a community-based survey conducted in the catchment area of the Dande Health and Demographic Surveillance System (Dande HDSS) [17], located in the Dande Municipality of Bengo Province, between 18 September 2013 and 28 March 2014. This study, named CardioBengo, constituted a larger survey of cardiovascular risk factors [18], and was based on the World Health Organization STEPwise approach to Surveillance (STEPS) to Chronic Disease Risk Factor manual (core and expanded version 3.0) [19].

### Sample characterization

The CardioBengo baseline survey was created and implemented with a representative sex- and age-stratified random sample of the Dande HDSS population aged 15–64 years as its basis [18]. Those aged 65 and older were not included because of their low representation in the general population (only 3.6% in the Dande HDSS) [17]. Younger participants (15–24 years) allowed a better representation of the demographic structure and created a stronger baseline for future surveys.

A total of 3,515 individuals were selected. After two failed attempts to contact them at their homes on different hours on weekdays, 1,025 were considered unreachable and six refused to participate, resulting in a final sample of 2,484 individuals. From these, 12 were excluded from the final analysis herein because of missing information on tobacco variables; thus, the final sample was 2,472.

### Study variables

Information on sex, age, education, family income, and alcohol consumption was collected through interviews [18,19] conducted by trained interviewers in Portuguese, and if necessary in the local language of Kimbundu. For analysis, age was categorized into five 10-year groups: 15–24, 25–34, 35–44, 45–54, or 55–64. Education was categorized by number of completed years of school: none, 1–4, 5–9, or 10 or more. Monthly family income was recorded in the Angolan kwanza, which then was converted into US dollars and categorized into: none, $150 or less, $151–299, or $300 or more. Frequency of consumption of alcoholic beverages during the preceding 12 months was recorded as: none, daily, 5–6 days/week, 1–4 days/week, or 1–3 days/month. Area of residence (urban or rural) was classified as described for the Dande HDSS area [17].

### Assessment of tobacco use and nicotine dependence

Subjects were asked if they currently use or had ever used any type of tobacco (smoked or smokeless), their age at initial use, current use, and average consumption. For analysis, individuals were classified as current, ex-, and never-users. Current users were asked about the type of tobacco used: manufactured, hand-rolled, and/or smokeless. Smokers (manufactured and/or hand-rolled cigarettes) were further grouped by frequency of consumption (≥1 and <6, ≥6 and <20, or ≥20 cigarettes/day).

A validated version of the Fagerström Test for Nicotine Dependence, in Portuguese [20], was also integrated into the interview. Per the test, nicotine dependence score was categorized as 0–2: very low; 3–4: low; 5: medium; 6–7: high; or 8–10: very high.

### Statistical analysis

Data were entered twice into a PostgreSQL (University of California, Berkeley, CA, USA) database and imported into IBM SPSS Statistics for Macintosh, Version 23.0 (IBM Corp., Armonk, NY, USA) for statistical analysis. All analyses were conducted considering the sampling weights [18]. Descriptive data are reported as absolute frequencies and percentages, and mean and standard deviation (SD) when appropriate. Prevalence of tobacco use and its 95% confidence interval (95% CI) were calculated according to the socio-demographic and behavioural characteristics.

### Ethics

All procedures performed in this study were in accordance with the standards of the 1964 Declaration of Helsinki and its later amendments. The Ethics Committee of the Angolan Ministry of Health approved the CardioBengo study protocol and all use of secondary data. Written informed consent was obtained from each participant or from the parent or legal guardian of those under 18 years old. A copy of the signed consent form, as well as contact information, was subsequently delivered to each participant.

## Results

As shown in Table 1, the great majority of the participants reported never using any type of tobacco (86.1% overall: 94.1% of females, 77.5% of males). The prevalence of current smoking was 6.1%, and was higher among males (10.0%) than females (2.6%), and the prevalence of ex-smokers was 7.5% (12.2% in males and 3.2% in females). Only five participants reported use of smokeless (chewing) tobacco (0.2%, 95% CI: 0.1–0.5).

**Table 1:** Prevalence, characteristics of tobacco use and nicotine dependence in smokers, by gender.

	Total (*n* = 2,472)		Female (*n* = 1,283)		Male (*n* = 1,189)	
	*n*	% (95% *CI*)*	*n*	% (95% *CI*)*	*n*	% (95% *CI*)*
**Tobacco use**						
Never	2,129	86.1 (84.7–87.4)	1,207	94.1 (92.6–95.2)	922	77.5 (75.1–79.8)
Smokeless	5	0.2 (0.1–0.5)	2	0.2 (0.0–0.6)	3	0.3 (0.1–0.7)
Current smokers	152	6.1 (5.3–7.2)	33	2.6 (1.8–3.6)	119	10.0 (8.4–11.8)
Ex-smokers	186	7.5 (6.5–8.6)	41	3.2 (2.4–4.3)	145	12.2 (10.5–14.2)
Uptake below the age of 18	113	34.7 (29.7–40.0)	27	39.7 (28.9–51.6)	86	33.3 (27.9–39.3)
**Daily smoked cigarettes (manufactured or hand-rolled)**						
≥1 and <6 cigarettes/day	99	65.1 (57.3–72.3)	28	84.8 (69.1–93.3)	71	59.7 (50.7–68.0)
≥6 and <20 cigarettes/day	36	23.7 (17.6–31.0)	4	12.1 (4.8–27.3)	32	26.9 (19.7–35.5)
≥20 cigarettes/day	17	11.2 (7.1–17.2)	1	3.0 (0.5–15.3)	16	13.4 (8.4–20.7)
**Nicotine Dependence (Fagerström Test)**^**a**^						
Very low dependence (0–2)	77	50.7 (42.8–58.5)	17	51.5 (35.2–67.5)	60	50.4 (41.6–59.2)
Low dependence (3–4)	50	32.9 (25.9–40.7)	13	39.4 (23.2–56.3)	37	31.1 (23.5–39.9)
Medium dependence (5)	16	10.5 (6.6–16.4)	3	9.1 (3.1–23.6)	13	10.9 (6.5–17.8)
High dependence (6–7)	8	5.3 (2.7–10.0)	0	-	8	6.7 (3.4–12.7)
Very high dependence (8–10)	1	0.7 (0.1–3.6)	0	-	1	0.8 (0.1–4.6)

* post-stratification weights used as described in the methods section.

^a^ only determine for cigarette smokers.

Around one-third of ever-smokers (current and ex-) began smoking before age 18 (Table 1). The average age at initiation was 20.1 (±6.9) years; 19.8 (±6.1) years for males and 21.4 (±9.3) for females. None of the current smokers reported intermittent use, with most reporting five or fewer cigarettes per day (84.8% of females, 59.7% of males) (Table 1). The average number of daily smoked cigarettes was 6.6 (±6.8), higher among males (7.5 ± 7.2) than females (3.4 ± 3.3).

The nicotine dependence level was rated very low or low in 83.6% of the smoker population. Only 9.1% of females presented a medium level and none were above, while 18.4% of males had a medium or higher level (Table 1). Table 2 shows a breakdown of the mean values of nicotine dependence by variable categories, revealing that males had consistently higher levels of dependence than females. Among males, younger individuals presented a slightly higher mean value, as with males from urban areas or who reported daily alcohol consumption. Among females, the highest values of mean nicotine dependence were found in rural areas and among women aged above 45 years (Table 2).

**Table 2:** Prevalence of smoking and ex-smoking by socio-demographic caracteristics and alcohol consumption frequency, by gender.

Sociodemographic characteristics	Female			Male		
	Current Smokers (*n* = 33)	Ex-Smokers (*n* = 41)	Nicotine Dependence (*n* = 33)	Current Smokers (*n* = 119)	Ex-Smokers (*n* = 145)	Nicotine Dependence (*n* = 119)
	% (95% *CI*)*	% (95% *CI*)*	Mean ± SD	% (95% *CI*)*	% (95% *CI*)*	Mean ± SD
**Age**						
15–24 years	-^a^	0.3 (0.0–1.4)	-^a^	3.4 (2.1–5.3)	5.8 (4.0–8.1)	3.0 ± 2.3
25–34 years	0.9 (0.3–2.6)	-^a^	2.3 ± 0.6	10.0 (7.2–13.9)	8.4 (5.8–12.0)	2.9 ± 1.9
35–44 years	2.5 (1.2–5.4)	3.8 (2.0–7.0)	1.6 ± 1.4	17.6 (12.5–24.3)	15.6 (10.8–22.0)	2.6 ± 1.9
45–54 years	6.8 (4.0–11.3)	7.9 (4.8–12.6)	2.7 ± 1.4	17.4 (11.6–25.1)	30.6 (23.1–39.3)	2.7 ± 1.8
55–64 years	9.9 (5.9–16.2)	13.0 (8.3–19.8)	2.4 ± 1.3	25.8 (18.1–35.3)	29.9 (21.7–39.6)	2.3 ± 1.9
**Place of residence**						
Rural	5.3 (19.8–31.0)	2.4 (1.1–5.2)	3.0 ± 0.9	25.0 (19.8–31.0)	15.4 (11.2–20.6)	2.3 ± 1.9
Urban	2.1 (5.3–8.5)	3.4 (2.4–4.7)	2.0 ± 1.5	6.8 (5.3–8.5)	11.4 (9.6–13.6)	3.0 ± 2.2
**Education**						
none	12.0 (8.2–17.1)	12.9 (9.0–18.1)	2.5 ± 1.4	29.4 (13.3–53.1)	18.8 (6.6–43.0)	2.0 ± 2.2
1–4 years	1.6 (0.8–3.2)	2.5 (1.4–4.4)	1.5 ± 2.6	33.1 (25.7–41.5)	21.2 (15.1–28.9)	3.2 ± 2.6
5–9 years	0.2 (0–1.2)	0.7 (0.2–1.9)	2.5 ± 2.1	11.3 (9.0–14.1)	13.7 (11.1–16.7)	2.4 ± 1.8
≥10 years	-^a^	0.6 (0.1–3.4)	-^a^	1.5 (0.7–3.1)	7.5 (5.4–10.2)	2.5 ± 1.4
**Montlhy Family Income**						
No income	5.0 (2.0–12.2)	5.0 (2.0–12.2)	1.6 ± 1.3	15.2 (6.7–30.9)	18.8 (8.9–35.3)	3.0 ± 2.0
≤150 USD	3.0 (1.7–5.1)	3.7 (2.3–6.0)	2.7 ± 1.6	14.9 (11.0–20.0)	18.6 (14.2–24.0)	2.1 ± 1.9
151–299 USD	1.9 (0.8–4.9)	2.9 (1.3–6.2)	1.4 ± 1.8	13.6 (9.4–19.3)	13.6 (9.4–19.3)	3.4 ± 2.3
≥300 USD	2.1 (0.4–11.1)	2.1 (0.4–11.1)	2.0 ± 0.0	10.0 (6.2–15.8)	16.7 (11.6–23.4)	3.0 ± 1.5
**Alcohol consumption**						
No	1.4 (0.8–2.4)	3.0 (2.1–4.4)	2.5 ± 1.3	5.2 (3.8–7.2)	10.4 (8.3–13.0)	2.7 ± 1.7
Yes	5.4 (3.5–8.1)	3.3 (2.0–5.6)	2.2 ± 1.3	17.2 (14.2–20.8)	15.0 (12.2–18.4)	2.6 ± 1.3
**Alcohol consumption frequency**						
Daily	16.0 (6.4–34.7)	4.2 (0.7–20.2)	1.8 ± 0.9	25.9 (16.1–38.9)	18.5 (10.4–30.8)	3.5 ± 2.6
5–6 days per week	4.2 (1.2–14.0)	2.1 (0.4–11.1)	1.5 ± 2.6	12.7 (8.3–18.9)	16.6 (11.5–23.3)	2.7 ± 2.0
1–4 days per week	4.6 (2.6–7.8)	3.4 (1.8–6.4)	2.4 ± 1.4	19.4 (14.9–24.7)	14.1 (10.3–19.0)	2.3 ± 1.8
1–3 days per month	7.3 (2.9–17.3)	5.4 (1.8–14.6)	2.2 ± 1.3	9.8 (4.3–21.0)	11.5 (5.4–23.0)	2.9 ± 2.2

* post-stratification weights used as described in the methods section.

^a^ No individuals in this category

As shown in Table 2, the prevalence of smoking was highest in the 55–64 age group for both sexes. The rural area had a higher smoking prevalence. Participants with 10 or more years of education had the lowest prevalence in both sexes. The prevalence was similar among income categories but slightly higher for those with lower levels of family income. Those who consumed alcohol (especially daily users) presented a higher prevalence of smoking. Similar trends were found for ex-smokers (Table 2).

## Discussion

The analysis of the results from this survey contributes to the small body of tobacco-use-related literature on Angola. Quite recently, the Global Burden of Disease Study (GBD) estimated a smoking prevalence of 1.6% in females and 14.2% in males for Angola in 2015 [6].

Without a national prevalence survey, only a subnational survey from Huambo Province was conducted under the auspices of the World Health Organization in 2010, and in a sample aged 13–15 years found a 2.3% prevalence of smoking (3.2% in boys, 0.3% in girls) [21]. Three other studies published since 2000 provided data on tobacco smoking: a survey of 667 adult students of health sciences in Lubango estimated a prevalence of 4.0% [22]; a study conducted on 615 active employees of the University Agostinho Neto, Luanda, found a value of 7.2% (10.2% in males, 4.4% in females) [23]; and in 1,464 individuals aged 25–64 years, surveyed in 2011 for hypertension in the same area as in the present study, the prevalence was 9.8% (18.3% in males, 4.3% in females) [24].

To integrate our results with the GBD estimation and these four studies, it was necessary to look at the age range of each. This survey included individuals aged 15–64 years, and none of the above surveys had such a wide age range. The two with younger populations—Huambo and Lubango [21,22]—had lower prevalence (2.3% and 4.0%, respectively), and the two with older populations—Luanda and Bengo [23,24]—were higher (7.2% and 9.8%, respectively). This situates the present findings in the middle, at 6.1%.

Smoking prevalence estimates present vast heterogeneity among countries in the sub-Saharan Africa region [25,26]. National estimates for those countries, mainly after STEPS surveys, ranged from 1.8% in Zambia to 37.7% in Sierra Leone [11,25,27]. In this way, the present results evidently place Angola low in prevalence rankings for the region.

A commonality between the present study and the rest of the Angola-based studies regarding tobacco is that the prevalence of current smoking has been consistently higher among males than females. Smoking is the preferred type of tobacco consumption for males when compared with females [26,28]. As in other populations, smoking among females was significantly less prevalent, across all socio-demographic characteristics [26–29]. Culturally, female smokers are not generally well accepted [29], and use of smokeless tobacco is more frequent among females than males in Sub-Saharan Africa [28]. However, social changes and economic growth in Angola may reflect females’ empowerment, including more equitable access to income and educational opportunities and, therefore, accompanied by shifts in societal suitability and acceptance of female smokers [29].

The rural areas have a higher proportion of smokers them the urban areas in this survey, while it is more common to encounter greater prevalence of smokers in urban areas of sub-Saharan African countries [27,30]. The region where the survey was conducted is only 60 km from the capital. It serves as a commercial post between the capital and the northern cities, and in this way may influence the publicity of tobacco in rural areas located near major roads to other provinces.

The relation between smoking and education level, with a higher prevalence of smokers among the less-educated, who may lack health literacy to critically evaluate their decisions regarding tobacco use [7,31], is common in other African surveys. Low levels of family income are usually associated with low levels of education, and indeed the prevalence of smokers was also higher among low-income individuals in the present study. This reveals a higher level of exposure among the population’s more disadvantaged groups, which further contributes to inequalities in health promotion.

Together with the economic difficulties that youth show as a reason for not smoking in Angola [14], the long period of colonial and civil war (1961–2002) may have strongly influenced the higher prevalence among older groups and the initiation age of the majority of the individuals. This evidence is reinforced by the prevalence of ex-smokers among older people and the duration of smoking habits that correspond with normal years of active military duty. This phenomenon of smoking, together with alcohol consumption and the use of other drugs among populations from regions with armed conflict is described in other studies, and often associated with rituals of manhood and integration in armed groups [32,33].

This connection associated with the higher prevalence of current and ex-smokers in the groups of less-educated and less-wealthy males and females must be acknowledged and further studied with qualitative approaches that enable understanding of the impact of war and post-traumatic stress on tobacco and alcohol consumption patterns in Angola. This accumulation of behavioural risk factors, which occurs in this population, must be a concern focused on in future interventions addressing addiction and health education.

With regard to addiction, the present results revealed that low nicotine dependence levels were still predominant in this population, with low mean numbers of cigarettes smoked daily (3.4 in females and 7.5 in males). The efficacy of individual smoking cessation strategies can be improved by considering the target group’s nicotine dependence level [34].

The low rates of smoking prevalence and nicotine dependence among smokers, and the substantial rate of ex-smokers, can be associated with individuals’ weighty economic difficulties and the end of the war period. Angola has never had a legal framework for tobacco control; this is now predicted in the national plan for health development for 2012–2025 [14]. There are also absolutely no cessation programs or health promotion initiatives that specifically target tobacco use. This owes to the inadequacy of health structures and lack of health professionals trained in this subject, as identified by the Ministry of Health [14].

### Strengths and limitations

The present study findings need to be interpreted cautiously, as the Dande HDSS was designed as a district-level surveillance system. Even if it reflects the national demographic structure of Angola in 2014 (young population with 50.3% aged 15–64 years, 51.5% females, and 68% concentrated in urban areas) [35], the goal herein was not to infer at a country level. This cross-sectional survey only covered a relatively short time period and could not capture possible cycles of temporary migration. Variables were also self-reported, which may have resulted in self-report bias and reflected social desirability. Additionally, information on real household constitution was not available, making the family income a possibly biased proxy of the participants’ economic power.

Migration and geographical isolation of some settlements within the Dande HDSS, together with the fact that working individuals were unavailable during the daytime [17], were reflected in the sampling definition, with a predictable 30% nonparticipation rate [18]. The distribution of non-respondents was uneven, with a higher proportion among younger individuals and males; this may have caused imbalance in the estimates in some strata.

However, this study is part of the largest community-based survey of NCD risk factors in Angola, as the first to address nicotine dependence in smokers in the general population. It is also expected to provide a baseline for further evaluating trends in tobacco use and understanding the dynamics in this population.

### Conclusions

This population, such as others in sub-Saharan Africa, is in the early stages of a possible tobacco epidemic, is characterized by low rates of smoking and increasing use of cigarettes among males [26,27]. However, and differently from high-income countries, the more educated and wealthier males do not seem to be those initially affected by the epidemic; the poorer and less-educated show higher smoking prevalence.

Tobacco use in Africa is on the rise as the tobacco industry shifts its marketing focus from the West to areas in Africa and Asia seen as having strong market growth potential [10,11,36]. Increasing urbanization and globalization, together with non-existent policies for tobacco control in Angola, and low product prices, enhance the susceptibility to higher levels of tobacco use in the near future.

Mass media campaigns and taxation of tobacco products, as proposed in the WHO Framework Convention on Tobacco Control [12,13,36], together with the expected implementation of tobacco control laws, may counteract the expected rising of smoking prevalence in Angola while tobacco use is still relatively low.
